# Comprehensive Reconstruction and Visualization of Non-Coding Regulatory Networks in Human

**DOI:** 10.3389/fbioe.2014.00069

**Published:** 2014-12-10

**Authors:** Vincenzo Bonnici, Francesco Russo, Nicola Bombieri, Alfredo Pulvirenti, Rosalba Giugno

**Affiliations:** ^1^Department of Computer Science, University of Verona, Verona, Italy; ^2^Laboratory of Integrative Systems Medicine (LISM), Institute of Informatics and Telematics (IIT) and Institute of Clinical Physiology (IFC), National Research Council (CNR), Pisa, Italy; ^3^Department of Computer Science, University of Pisa, Pisa, Italy; ^4^Department of Clinical and Experimental Medicine, University of Catania, Catania, Italy

**Keywords:** microRNAs, lncRNAs, non-coding RNAs, networks, cytoscape, gene expression

## Abstract

Research attention has been powered to understand the functional roles of non-coding RNAs (ncRNAs). Many studies have demonstrated their deregulation in cancer and other human disorders. ncRNAs are also present in extracellular human body fluids such as serum and plasma, giving them a great potential as non-invasive biomarkers. However, non-coding RNAs have been relatively recently discovered and a comprehensive database including all of them is still missing. Reconstructing and visualizing the network of ncRNAs interactions are important steps to understand their regulatory mechanism in complex systems. This work presents *ncRNA-DB*, a NoSQL database that integrates ncRNAs data interactions from a large number of well established on-line repositories. The interactions involve RNA, DNA, proteins, and diseases. ncRNA-DB is available at http://ncrnadb.scienze.univr.it/ncrnadb/. It is equipped with three interfaces: web based, command-line, and a Cytoscape app called ncINetView. By accessing only one resource, users can search for ncRNAs and their interactions, build a network annotated with all known ncRNAs and associated diseases, and use all visual and mining features available in Cytoscape.

## 1 Introduction

After the sequencing of the human genome, it became evident that only 20,000 genes are protein-coding, while over 98% of all genes are untranslated non-protein-coding RNAs (ncRNAs) (ENCODE Project Consortium, 2012). During the last years, thousands of ncRNAs have been identified in the eukaryotic transcriptome (Khalil et al., 2009; Bu et al., 2011). Usually, ncRNAs are divided into two groups according to their length: short ncRNAs, consisting of <200 nucleotides, and long non-coding RNAs (lncRNAs), whose size ranges from 200 nucleotides up to 100 kb (Mattick, 2001).

The microRNAs (miRNAs) family is the best known class of short ncRNAs. They regulate gene expression and contribute to development, differentiation and are responsible of carcinogenesis. The aberrant expression or alteration of miRNAs also contributes to many of human pathologies, including cancer (Lu et al., 2005). Moreover, a significant amount of miRNAs has been found in extracellular human body fluids (Mitchell et al., 2008; Hanke et al., 2010) and some circulating miRNAs in the blood have been successfully revealed as biomarkers for several diseases including cardiovascular malfunctions (Gupta et al., 2010b) and cancer (Mitchell et al., 2008).

An emerging class of ncRNAs consists of lncRNAs (Fatica and Bozzoni, 2014) They are both nuclear and cytoplasmic. Nuclear lncRNAs function by guiding chromatin modifiers to specific genomic loci (Rinn and Chang, 2012; Batista and Chang, 2013; Guttman and Rinn, 2012; Khalil et al., 2009; Tay et al., 2011) while many others have been identified in the cytoplasm (Batista and Chang, 2013). These lncRNAs are involved in gene regulation and often show sequence complementarity with transcripts that originate from either the same chromosomal locus or independent loci.

One of the most recently discovered and not yet functionally characterized class is the circular RNA (circRNAs) (Memczak et al., 2013) Numerous circRNAs form by head-to-tail splicing of exons, suggesting previously unrecognized regulatory potential of coding sequences. Recent results (Memczak et al., 2013) have shown that thousands of well-expressed stable circRNAs have both tissue and developmental-stage specific expression. Moreover, human circRNAs are bound by miRNAs such as the miR-7 showing a potential role of circRNAs as post-transcriptional regulators.

Understanding the complex system derived from the interactions of regulators and possible targets gives a clue on the dynamics and causes of disorders (Couzin, 2007). In this direction, platforms to visualize networks such as Cytoscape (Shannon et al., 2003) together with tools to visualize and analyze them are becoming crucial in systems biology studies.

miRScape (Ferro et al., 2009) is one of the first Cytoscape plug-in visualizing protein–protein interaction networks annotated with miRNAs. It uses a web knowledge base (Laganà et al., 2009) to infer associations between genes and phenotypes though miRNAs. CyTargetLinker (Kutmon et al., 2013) is a recent Cytoscape app that builds biological networks annotated with miRNAs, transcription factors, and drugs.

Several methodologies are designed to analyze the regulatory effect of miRNAs and transcription factors in protein-coding genes (Liu et al., 2009, 2014; Sales et al., 2010; Huang et al., 2011; Laczny et al., 2012; Le et al., 2013; Guo et al., 2014). Some of them export the results also in a Cytoscape network format. For example, Magia (Sales et al., 2010) allows to perform statistical analysis on miRNAs and gene expressions. TSmir (Guo et al., 2014) browses regulatory network of tissue-specific miRNAs with transcriptor factors. mirConnX (Huang et al., 2011), given a network of genes, transcriptor factors, and miRNAs, extends it with further TF and miRNA–gene intersections inferred by user expression data. miRTrail (Laczny et al., 2012) analyzes the role of miRNAs and genes deregulated in a disease by using a miRNA-gene networks and expression data.

In this work, we have imported and integrated associations among non-coding RNAs (miRNAs, circulating miRNAs, lncRNAs, and other non-coding), genes, RNAs, and associated diseases from 10 on-line databases. The database, named non-coding RNA Human Interaction Data Base (ncRNA-DB), is built on top of the NoSQL platform OrientDB. It is kept updated by common semi-automated procedures. The interaction data of ncRNA-DB can be simply searched and visualized by a web based or a command-line interface. The database is accessible through a Cytoscape app, called ncINetView, which allows to: (i) build a network annotated with all known ncRNAs and associated diseases by accessing to only one database, and (ii) use all visual and mining features available in Cytoscape app store to analyze it. At http://ncrnadb.scienze.univr.it/ncrnadb/, users can search in ncRNA-DB, export the results in text format, download the command-line interface, Java API, the app ncINetView, and use ncRNA-DB as server for third party client applications.

## 2 Construction and Content

### 2.1 Data source

Non-coding RNA human interaction data base integrates data from several state of the art non-coding databases. We selected sources that cover the majority of non-coding RNAs information with high quality and updated data. Moreover, this first version of ncRNA-DB focuses on databases of known interactions between non-coding RNAs and mRNAs. We discarded non-coding RNAs with unknown interactions such as piRNAs (RNA Piwi-interacting). In the following subsections, we give an overview of data sources in ncRNA-DB. Table 1 summarizes the numbers of integrated data and how many are shared among datasources.

**Table 1:** **The number of imported elements from external resources and how many among them are present at least in another datasource**.

DataSource	Number of entities	Shared
CIRC2TRAITS	83,432	326
HMDD.2	8,040	282
LNCRNADISEASE	1,505	244
MIRANDOLA.1.6 2246	98	
NPINTER.2.0	138,328	440
MIRTARBASE	40,532	218
STARBASE.V2.0	31,463	8

*This representation of shared notation is dictated by the fact that the number of elements shared in three or more datasources is approximately close to 0*.

#### 2.1.1 Nomenclature of non-coding RNAs

In ncRNA-DB, we used The HUGO Gene Nomenclature Committee (HGNC) as official database of approved names and aliases. HGNC is responsible for approving unique symbols and names for human loci, including protein-coding genes, ncRNA genes, and pseudogenes, to allow unambiguous scientific communication (Gray et al., 2012)^1^.

#### 2.1.2 Long non-coding RNAs databases

In this work, we selected several lncRNAs databases that provide a central repository of known lncRNAs, their aliases, and published characteristics. lncRNAdb (Amaral et al., 2011) is one of them and it is available online at http://www.lncrnadb.org.

Another database is The LncRNADisease (Chen et al., 2013)^2^. It is a resource for the experimentally supported LncRNA-disease association data. The platform integrates also tools for predicting novel LncRNA-disease associations. Moreover, LncRNADisease contains lncRNA interactions at various levels, including proteins, RNAs, miRNAs, and DNA.

We also included general non-coding databases such as NONCODE^3^, which is a database of all kinds of non-coding RNAs (except tRNAs and rRNAs) containing 210,831 lncRNAs of several species (Bu et al., 2011).

#### 2.1.3 Circular RNAs database

Circ2Traits^4^ is a comprehensive database for circRNA potentially associated with diseases and traits (Ghosal et al., 2013) circRNAs, formed by covalent linkage of the ends of a single RNA molecule, are newly discovered RNAs that sponge miRNAs to block their function (Memczak et al., 2013). Circ2Traits uses the circRNA dataset from Memczak et al. (2013). This dataset consists of 1,953 predicted human circRNAs along with their genomic coordinates, annotation, and predicted miRNA seed matches. The disease related miRNA data are taken from miR2disease (Jiang et al., 2009). The authors collect the miRNA–mRNA interaction data predicted by miRanda (Betel et al., 2008), TargetScan (Lewis et al., 2005), PiTA (Kertesz et al., 2007), PicTar (Krek et al., 2005), and RNA22 (Loher and Rigoutsos, 2012). Moreover, a dataset of predicted miRNA and lncRNA interaction pairs is collected from the miRCode database (Jeggari et al., 2012).

#### 2.1.4 microRNA databases

Non-coding RNA human interaction data base includes The Human microRNA Disease Database (HMDD) (Li et al., 2013), a database of curated experiment-supported evidence for human miRNAs and disease associations^5^. The database contains detailed and comprehensive annotations of human miRNA-disease associations, including those from the evidence of genetics, epigenetics, circulating miRNAs, and miRNA-target interactions.

Another important resource is the miRandola database (Russo et al., 2012, 2014)^6^. It is a manually curated database of extracellular circulating miRNAs. It is a comprehensive classification of different extracellular miRNA types and a collection of non-invasive biomarkers for several diseases (e.g., cancer and cardiovascular diseases).

#### 2.1.5 Interaction databases

We included several sources for non-coding RNAs interactions. The miRTarBase database (Hsu et al., 2014)^7^ provides experimentally validated miRNA-target interactions.

NPInter (Wu et al., 2006)^8^ reports functional interactions between non-coding RNAs (except tRNAs and rRNAs) and biomolecules (proteins, RNAs, and DNA), which are experimentally verified. The authors collected primarily physical interactions, although several interactions of other forms are also included. Interactions are manually collected from publications, followed by an annotation process that uses known databases including NONCODE (Bu et al., 2011), miRBase (the miRNA registry) (Kozomara and Griffiths-Jones, 2013), and UniProt (the database of proteins) (UniProt Consortium, 2013).

starBase (Li et al., 2014)^9^ reports RNARNA and proteinRNA interactions from 108 CLIP-Seq (PAR-CLIP, HITS-CLIP, iCLIP, and CLASH) about 37 independent studies. The database contains about 9,000 miRNA–circRNA, 16,000 miRNA–pseudogene, and 285,000 protein–RNA relations. It also contains predicted miRNA–mRNA and miRNA–lncRNA interactions.

### 2.2 Data schema

#### 2.2.1 ncRNA-DB identifier

Public databases catalog biological entities (e.g., ncRNAs) via nomenclatures. They can be human readable names or alphanumeric identifiers. For example, genes are classified by their names, their symbols, or database-specific identifiers. For example, the *breast cancer 1* gene can be identified by its assigned symbols BRCA1, BRCC1, and PPP1R53, or by its specific database identifiers like HGNG:1100, Entrez Gene 672, and UCSC uc002ict.3.

Non-coding RNAs have been relatively recently discovered and a comprehensive database including all of them is still missing. The non-coding RNAs knowledge is spread among several databases and ambiguity on the identifiers exists. Moreover, new discovered entities are named with internal identifiers and they are not reported in any other databases. This is the case for example of NONCODE v4, the largest collection of ncRNAs available on-line, where most of the reported ncRNAs can be only mapped to NONCODE.

In ncRNA-DB, we use a generic resource identifier system (named RID) together with a unique system-scope identifier assigned by OrientDB (called ORID).

The RID is composed by three parts (or levels) *EntityType: DataSource: Alias.name*. The EntityType indicates the biological classification of the element such as ncRNA, RNA (not including ncRNA), Gene, Disease, and Others for all other cases including entities with unspecified type in the original data source. The DataSource reports the name of the external data source from where we got the data together with its version (e.g., HMDD_2). The Alias.name represents the nomenclature used in the data source.

#### 2.2.2 Graph database schema for ncRNA-DB

A set of biological entities (genes, ncRNAs, RNAs, and diseases) and their relations (physical interactions, functional relations, and so on) can be modeled as a graph, a mathematical object composed by nodes (entities) and edges (relations).

Relational database management systems (RDMS) are widely used to store biological data. However, new rising models, grouped under the name of NoSQL (Not only SQL) databases (Stonebraker, 2010; Han et al., 2011), are becoming quite popular for web and biological applications. They can provide schema-less representation for non-structured data and can be easily implemented in a distributed fashion resulting effective for Big Data problems (Cattell, 2011).

NoSQL system can be classified into four classes, even if some of them belongs to more than one class: (i) column model, where data are represented by tuples, (ii) document-oriented databases for storing, retrieving, and managing document-oriented information, also known as semi-structured data, (iii) key-value store, where data are stored as a collection of key-value pairs stored using associative arrays, maps, symbol tables, or dictionaries, and (iv) graph databases, where data are modeled using a graph structure. These often implement the object-oriented model by modeling concepts like classes, instances, inheritance, and polymorphism.

Non-coding RNA human interaction data base is implemented in OrientDB (Tesoriero, 2013) OrientDB is both a graph model and an object-oriented model, on top of a document model. We chose OrientDB since it is a graph database and its object-oriented concepts are suitable to model the ncRNA-DB data. Furthermore, the use of OrientDB allows to give public accesses to our server, effective management of user privileges, use graph traversal procedures, and language bindings among a large choice. It offers a SQL-like interface in addition to several language specific interfaces. It is developed in Java and provides native Java API (Application Programing Interface) for accessing the database, which is suitable for developing Cytoscape applications.

**Figure 1:**
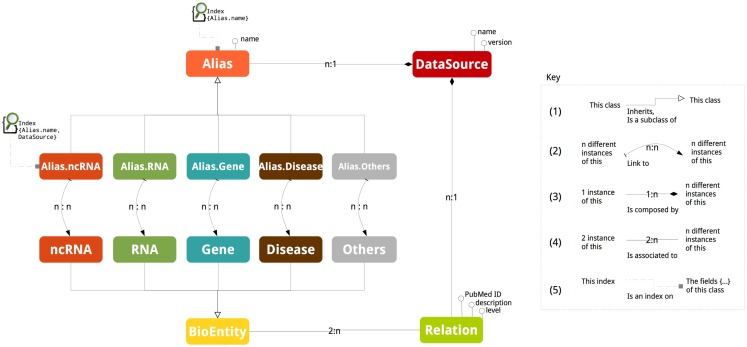
**The database schema**. The picture reports all the stored information together with their associations.

Figure 1 depicts the schema of ncRNA-DB. The abstract class *BioEntity* represents biological entities and it is specialized in the five sub-classes: ncRNA, RNA, Gene, Disease, and Others. Aliases are represented by the abstract class *Alias*, which is specialized in five different sub-classes related to the five entity types. DataSource is a class containing the external resource name and version from where the data are got or equivalently the official repository of the entity (e.g., NONCODE v4). An instance of a class is a particular value (e.g., realization, element, and data). In a graph model, instances of classes and sub-classes are nodes.

Class inheritance happens when a class is a specialization of the other one (Figure 1 mark 1). The naming of a biological entity by an alias is represented by adding an edge between the corresponding graph nodes. Due to the ambiguity of nomenclatures, these edges are *n*:*n* cardinality (Figure 1 mark 2). This means that, for example, an ncRNA can have different aliases and the same alias can refer to different ncRNAs.

Interactions among entities are modeled through a class called *Relation* associated to the class *BioEntity* (Figure 1 mark 3). The cardinality of the association is *n*:2, since an entity participates at more than one relation and a relation involves exactly two entities.

The attributes of *Relation* are the *PubMed ID* containing the reference of article reporting such relation, the *description* with the support sentences and *level* to store the interaction level. The interaction level indicates the molecular strata where the interaction is realized. This is represented by a pair of strings (*a *− *b*) with *a* and *b* belonging to (RNA, DNA, Protein, TF). For example, RNA–TF specifies that the ncRNA is interacting with the transcription factor of the gene; (RNA–DNA) indicates that the ncRNA is interacting with the coding genomic region of the gene; (RNA–Protein) describes that the ncRNA is interacting with the protein structure; and (RNA–RNA) tells that ncRNA is interacting with the transcript RNA. If the same relation is stored in two (or more) distinct data sources, two (or more) interaction edges are stored into our system. This choice is motivated by reporting for each interaction specific information such as the support sentence. The *level* is the string NA when this detail in the resource is not given.

When a class contains as field values of another class we indicate that a composition relation exists (Figure 1 mark 4). For example, a data source name and version is part of a RID, which represents an Alias. The *Relation* has a composition association with *DataSource* to external databases reporting it. The cardinality of composition relation is *n*:1 since an alias or a relation is reported in a data source and a data source contains more than one relation or alias.

Aliases act as access points to the data and they are indexed (Figure 1 mark 5). The abstract class *Alias* is indexed by a single field not-unique map on the element nomenclature (the third field of the RID, Alias.name). This is used when the search is performed by giving only the nomenclature. The *Alias.type* sub-classes are indexed by a composite key dictionary working on the second and third field of the RID, DataSource, and Alias.name. This index works when both the EntityType and the nomenclature are specified.

#### 2.2.3 Data import

Here, we give details on the imported data from each resource. ncRNA-DB integrates data concerning only *Homo sapiens*.

- HGNC: we imported a list of non-coding RNAs and their approved aliases used by other datasources, protein-coding genes, pseudogenes, and phenotypes (considered as diseases).
- lncRNAdb: we imported a list of non-coding RNAs and their aliases.
- circ2traits: we imported a set of interacting lncRNAs, circRNAs, and messenger RNAs together with the associated diseases and the PubMed IDs of articles where the interactions are reported.
- HMDD: we imported a list of diseases, the set of genes that interact with ncRNAs, PubMed IDs of articles together with the support sentences. Here, interactions are listed as ncRNA-disease or ncRNA-gene-disease. We split the multi-relation ncRNA-gene-disease into two distinct relations ncRNA-gene and ncRNA-disease.
- LncRNAdisease: we imported a list of lncRNAs, their aliases, associated diseases, interaction levels, PubMed IDs of articles supporting the interactions, and sentences describing details such as the type of dysfunction.
- Mirandola: we imported a set of miRNAs, their aliases, PubMed IDs of articles together with the support sentences.
- miRTarBase: we imported a set of miRNAs, their validated targets, and their aliases, PubMed IDs of articles together with the support sentences.
- NONCODE: we imported a list of non-coding RNAs, their aliases and a mapping of NONCODE into external identifiers.
- NPInter: we imported a set of ncRNAs, their interactions, interaction levels, PubMed IDs of referencing articles, and supporting sentences.

From the integrated data source files, we extracted the following fields: source, target, and interaction details such as interaction levels, reference papers, and support sentences. The main issues about importing data from several resources are aliases disambiguation and the missing of entity type classification. In a first phase, we extracted and combined from HGNC, NONCODE, and LNCRNADB the sets of aliases for each bioentity. At the end of this step, each bioentity will have some aliases uniquely assigned to it, and some others shared with other entities. In a second phase, for each entity we merge its aliases with those taken from all other datasets integrated in ncRNA-DB. Table 2 summarizes the number of aliases taken from the integrated datasources and how many are shared among them.

**Table 2:** **The total number of aliases associated with the imported elements from external resources and how many among them are present at least in another datasource**.

DataSource	Number of aliases	Shared
HGNC	436,361	19,368
NONCODE.V4	327,099	5,671
LNCRNADB	218	115
CIRC2TRAITS	16,730	1,076
HMDD.2	1,376	1,376
LNCRNADISEASE	1,366	285
MIRANDOLA.1.6	1,231	1,231
NPINTER.2.0	7,678	4,857
MIRTARBASE	62,207	12,998
STARBASE.V2.0	5,298	3,747

When the entity type of interaction actors are not provided, but only the entity levels (i.e., RNA-protein), we first searched the elements in the ncRNA-DB. If they were not present in any sub-classes (*ncRNA*, *ncRNA*, *RNA*, *Gene*, or *Disease*), we labeled them as Others.

At the end of the described ETL (Extract, Transform, and Load) procedure, we had: 853,543 alias, a total of 222,970 biological entities, 889,675 edges connecting *Alias* and *BioEntity* classes, and 238,524 entity relations.

Table 3 gives the total number of imported biological entities, grouped by type, and how many of them are actually involved in relations. Table 4 reports the number of ncRNAs interacting with other ncRNA-DB biological entities.

**Table 3:** **For each biological entity type we report the number of entries present in ncRNA-DB**.

Entity	Total	In relation
ncRNA	193,440	25,463
RNA	4,962	4,962
Gene	19,271	12,265
Disease	1,330	735
Others	6,700	5,517

*We report also the number of entities having relations with some other entities (details are given in Table 4)*.

**Table 4:** **The number of ncRNAs interacting with other ncRNA-DB biological entities**.

Relation	Total
ncRNA-ncRNA	77,982
ncRNA-RNA	36,369
ncRNA-gene	52,611
ncRNA-disease	16,662
ncRNA-others	132,663

## 3 Utility

OrientDB is supported by several language connectors, beside the native Java API. The user can query the system through programing language binding, or by using the OrientDB SQL-like console. It also implements technology standard like HTTP REST/JSON, TinkerPop Blueprints (for graph computing), and JDO (Java Data Object for object persistence). The user can develop software as client connected to the ncRNA-DB database.

Non-coding RNA human interaction data base is equipped with three alternative interfaces: (i) a CytoScape (version 3) app for importing data in a network visualization environment; (ii) a web interface; and (iii) a command-line interface for raw resource queries. Entities are specified by using their alias, through full or partial ncRNA-DB identifiers (RID or ORID).

The CytoScape plug-in and the command-line applications can be downloaded from the ncRNA-DB website at http://ncrnadb.scienze.univr.it/ncrnadb/. The documentation is also provided.

**Figure 2:**
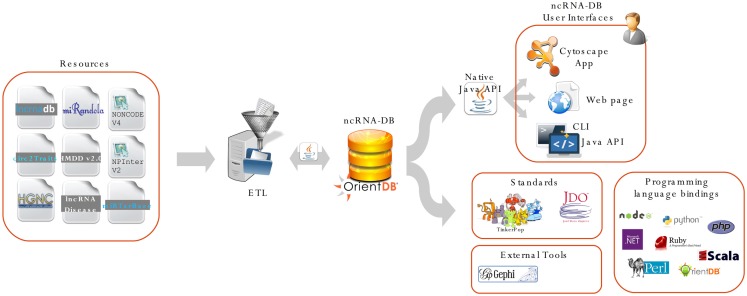
**We report the main architecture of our system**. The resource is integrated through an import procedure and stored into OrientDB. All the data are exposed using three different user interfaces: (i) the ncINetView; Cytoscape app; (ii) the web app; and (iii) the command-line interface. All the data can be also queried using languages APIs and third party applications. ncRNA-DB is designed to be a server for client applications, thanks to the features offered by OrientDB.

Figure 2 shows a complete schema of the proposed system, from the import phase to the user interfaces.

### 3.1 Cytoscape interface

The CytoScape app interface, ncINetView, allows users to: (i) annotate an existing network with the ncRNA-DB relations; and (ii) search ncRNA-DB relations of specific elements and to add them to a user network or to create a new network. The source code of the Cytoscape interface, ncINetView, is available at https://code.google.com/p/ncrnadb/.

#### 3.1.1 Add edges

The Add Edges takes an user network as input and annotates it with ncRNA-DB relations among its nodes.

The user selects the name of a network to be annotated by clicking on Network. The network needs to be already imported into the Network View of Cytoscape. In order to expand a subset of such a network, the user selects the relative nodes in the Network View Section and checks the Selected only option. The network table may have two columns specifying the biological entity type of each node together with the set of known aliases. The user assigns such columns to Type column and Alias column. The app maps each node of the network with the entities of the ncRNA-DB having the associated aliases.

The type column is optional. If missing, ncINetView creates one and associates the types in ncRNA-DB of the matched entities. The type of a vertex may be *ncRNA*, *RNA*, *Gene*, *Disease*, and *Others*. When it is labeled as Others, the user may assign a miscellaneous of entity types to the corresponding table entries or may leave it empty. Even in this case, the app tries to map all the matching aliases entities to the node. This behavior allows the user to specify nodes representing entities groups and to do disambiguation at a network data representation level.

The user can decide whenever some of the above entity types have to be excluded from the mapping. This can be done by unflagging the corresponding entity type check-boxes.

Once the user clicks on Import, the application retrieves from ncRNA-DB the matching biological entities and their relations. Then the user maps them into the network nodes and adds all found relations among them.

If the Include neighbors check-box is flagged, then the application retrieves all the ncRNA-DB neighbors of the matching entities and adds them to the mapped nodes, as well as relations among them and the other retrieved entities.

**Figure 3:**
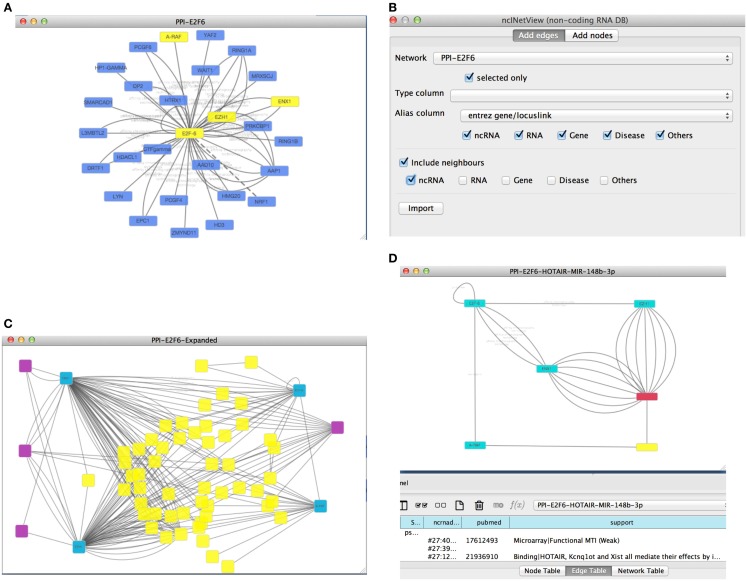
**(A)** The picture reports the retrieved Biogrid network (with the E2F6 query) with four nodes (E2F6, EZH1, EZH2, and ARAF) selected by the user; **(B)** the first panel of ncINetView allows the annotation of selected nodes with all ncRNA neighbors and all their interactions with actors of the network present in ncRNA-DB; **(C)** the resulted annotated network; **(D)** an extracted circuit by the user involving the long non-coding RNA HOTAIR, the selected genes and the hsa-mir-148p-3b. The presence of more than one edge connecting nodes is motivated by reporting in the table panel for each interaction specific information such as the support sentence.

As an example, we can retrieve a protein–protein interaction network from Biogrid using the proper Cytoscape option. We searched for the protein E2F6 and we retrieved all the known experimental validated interactions stored in Biogrid. To uncover potential novel important interactions, we focused on a subnetwork by selecting some protein-coding genes: E2F6, EZH1, EZH2, and ARAF (see Figure 3A). Next, we used our app to extend the network with non-coding RNAs (e.g., lncRNAs and miRNAs). This yielded a new network (see Figures 3B,C).

From all retrieved interactions, we analyzed those involving one lncRNA (HOTAIR, Gupta et al., 2010a) and one miRNA (miR-148b-3p) (see Figure 3D). The hypothesis for this kind of interactions could be the following: (1) the regulation of cell cycle and (2) the role of this circuit in the chromatin remodeling. In fact, it is well known that EZH1 and EZH2 (also called ENX1) are involved in the chromatin remodeling (Margueron et al., 2008). Moreover, these genes are up-regulated in several cancers and in particular EZH2 interacts with E2F6 contributing to cellular proliferation and cell cycle progression (Attwooll et al., 2005). Interestingly, the long non-coding HOTAIR is also involved in the chromatin remodeling, carcinogenesis and metastasis (Gupta et al., 2010a). HOTAIR over-expression is associated with the reprograming of the Polycomb complex PRC2 function in breast cancer (Gupta et al., 2010a) and colorectal cancer (Kogo et al., 2011). Furthermore, its up-regulation may be a critical element in metastatic progression. In this context, the miR-148b-3p is considered a tumor suppressor miRNA, and it is down-regulated in several cancers such as the colorectal cancer (Song et al., 2012). Moreover, it has been reported that the over-expression of miR-148b could inhibit cell proliferation *in vitro* and suppress tumorigenicity *in vivo* (Song et al., 2012). A possible mechanism of the tumorigenesis in colorectal cancer and other cancers, could act through the above molecules in a circuit, which involves the up-regulation of the cited proteins and the down-regulation of miR-148b-3p mediated by the lncRNA HOTAIR. In this case, HOTAIR may function as competing endogenous RNAs (ceRNAs) to sponge miR-148b-3p, thereby modulating the de-repression of its targets (e.g., ARAF, a proto-oncogene may involved in cell proliferation).

#### 3.1.2 Add nodes

Add Nodes allows users to search for biological entities by specifying their aliases.

**Figure 4:**
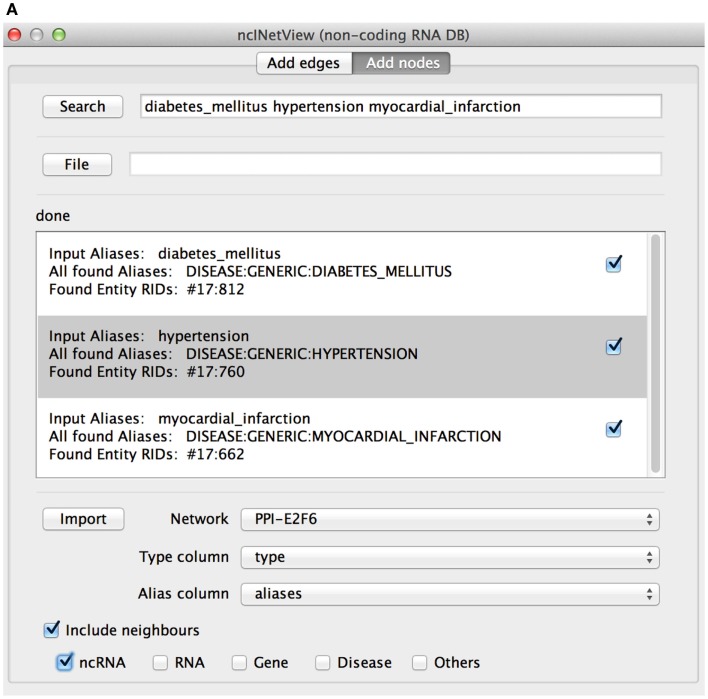

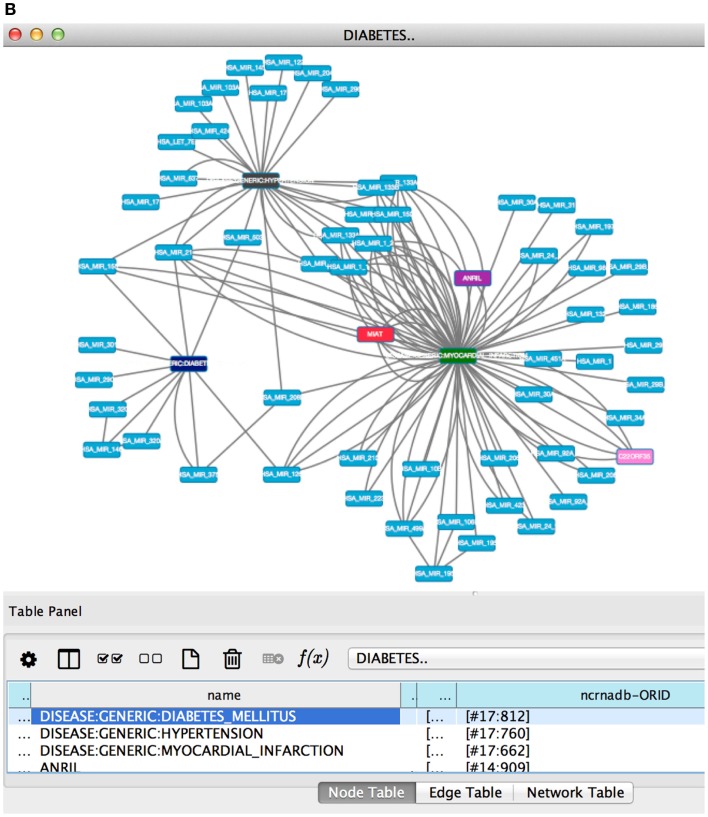
**The add nodes panel of ncINetView**. **(A)** The user performs a query specifying: diabetes mellitus, hypertension, and myocardial infarction. All elements found in ncRNA-DB are reported in the text area with the associated aliases. A check box is used to include the elements in the network generated by clicking in Import. User selects in Network among those present in the cytoscape network panel, which network must be annotated; together with the name of table columns containing the aliases and the type of each node (the last is optional). **(B)** The corresponding network view is generated.

In Search, the user specifies the entity nomenclatures to be searched separated by space (see Figure 4A). The app creates a node for each retrieved element. Aliases can be loaded also from file (File). The file has one or more aliases per row and each row corresponds to a node. If a row contains more elements than the node is a group node (i.e., a miscellaneous of entity types).

The app retrieves the matching entities and shows them in the Results panel (see Figure 4B). For each entity, the list of corresponding aliases and their biological types are shown. Users can select the entities to be imported in the network (Import).

In Network, the user selects the name of the network to be annotated among those available in the Cytoscape Network View. Furthermore, the user specifies which column of the network table should be assigned to Type column and Alias column that contains the entity type of the nodes and their aliases. The network can be also empty.

If the Include neighbors check-box is flagged, then the application retrieves all the ncRNA-DB neighbors of the matching entities and adds them to the mapped nodes. The user can decide whenever some neighbor types have to be excluded from the mapping. This can be done by unflagging the corresponding entity type check-boxes.

For example, let’s search for the diabetes mellitus, hypertension, myocardial infarction, and let’s get all non-coding RNAs associated with them (see Figures 4A,B). Several ncRNAs are associated to one, two, or all three diseases.

### 3.2 Web interface

**Figure 5:**
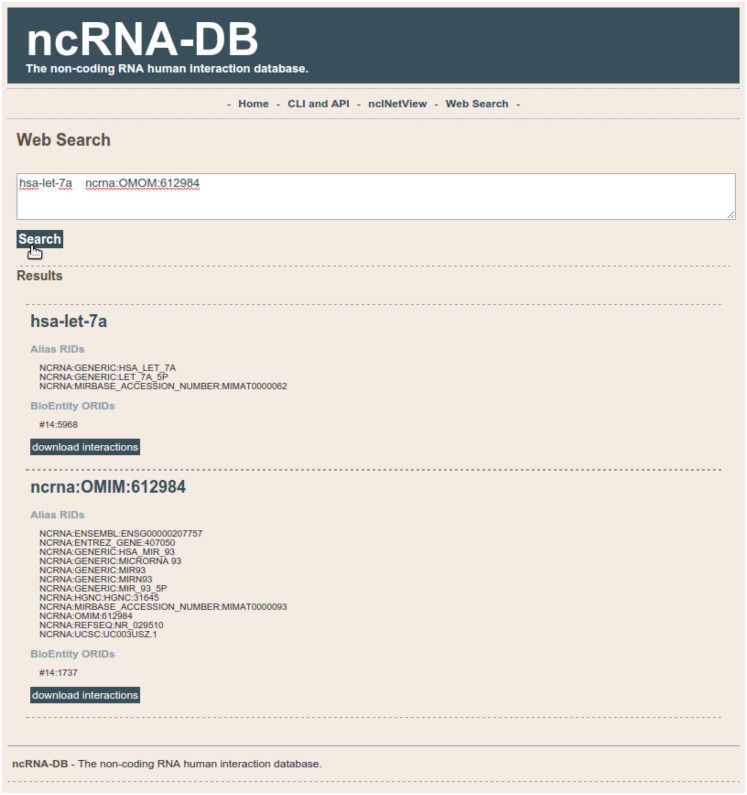
**The search section of the web interface of ncRNA-DB**.

We developed a web app for querying our database^10^. Users can search through a text area by putting a list of elements. The system will show the matching ncRNA-db entities and their neighbors (see Figure 5). Results can be saved in text format.

### 3.3 Command-line interface

We developed a command-line interface to ncRNA-DB for entity searching and relation retrieval. It is released as a Java package to be platform independent and it does not require any external dependency. It provides two commands for accessing data. The search command takes a list of aliases as input and returns the matching biological entities stored in ncRNA-DB. This command is also useful to verify whether an identifier is included in the database and to retrieve all its alternative nomenclatures. The second command, relations, receives a list of entities as input, and returns the relations between them stored in ncRNA-DB and their support information. The released package also provides Java API implementing the functionality described above. The documentation is provided as JavaDoc at ncRNA-DB web site. Alternately, users may adopt the GraphAPI of OrientDB. The source code of CLI interface is available at https://code.google.com/p/ncrnadb/.

## 4 Conclusion

In this paper, we have presented ncRNA-DB, an integrated database storing knowledge concerning ncRNAs, genes, and associated diseases. The system has been implemented within the NoSQL database OrientDB. It stores data coming from several leading resources such as HGNC, lncRNAdb, circ2Traits, HMDD, lncRNADiseases, miRandola, miRTarBase, NON-CODE, and NPInter. ncRNA-DB can be queried trough three interfaces. A Cytoscape App, named ncINetView, allows to annotate biological networks with ncRNA knowledge. A web app and a command-line interface, which allows users to query the ncRNA-DB and to extract information in a text format. The aim of the proposed system is to give a comprehensive access to all the knowledge available in the literature concerning ncRNAs and associated diseases. As a key characteristics, the integrated data aim to reduce the problem of different nomenclatures used by different sources. The ncRNA-DB is available at http://ncrnadb.scienze.univr.it/ncrnadb/.

## Conflict of Interest Statement

The authors declare that the research was conducted in the absence of any commercial or financial relationships that could be construed as a potential conflict of interest.

## Footnotes

^1^http://genenames.org

^2^http://210.73.221.6/lncrnadisease

^3^http://www.noncode.org/

^4^http://gyanxet-beta.com/circdb/

^5^http://www.cuilab.cn/hmdd

^6^http://atlas.dmi.unict.it/mirandola/

^7^http://mirtarbase.mbc.nctu.edu.tw/

^8^http://www.bioinfo.org/NPInter/

^9^http://starbase.sysu.edu.cn/

^10^http://ncrnadb.scienze.univr.it/ncrnadb/
